# Phylogenetic Conservatism and Ambient Temperature Shape Spatial Variation in Bat Occupancy and Species Richness Along a Subtropical Elevational Gradient

**DOI:** 10.1002/ece3.71912

**Published:** 2025-09-25

**Authors:** Carlos Henrique Russi, Selvino Neckel‐Oliveira, Vítor Carvalho‐Rocha, Carlos A. Peres

**Affiliations:** ^1^ Instituto Nacional de Pesquisas da Amazônia Programa de Pós‐Graduação em Ecologia Manaus Brazil; ^2^ Programa de Pós‐Graduação em Ecologia, Departamento de Ecologia e Zoologia Universidade Federal de Santa Catarina Florianópolis Santa Catarina Brazil; ^3^ School of Environmental Sciences University of East Anglia Norwich UK; ^4^ Instituto Juruá Manaus Brazil

**Keywords:** Chiroptera, functional ecology, habitat filtering, niche selection, occupancy models, phylogenetic comparative methods

## Abstract

Steep elevational gradients are natural laboratories for understanding species–environment relationships, as they provide high environmental heterogeneity over accessible distances. If species can colonize all available environments, their distributions become mainly a product of their niches, offering a unique opportunity to study these dynamics. Here, we capitalized on this ideal scenario to test whether ecological traits and evolutionary history interact with environmental variables to shape the occupancy of 27 bat species along a ~1300 m elevational gradient in subtropical Brazil. Using a multi‐species modeling approach, we integrated data from mist‐netting and acoustic recorders, environmental variables, ecological traits, and phylogeny to generate estimates that represent the entire bat assemblage. We found that most bat species in our study area are restricted to low elevations, with only two high‐elevation specialists. Ecological traits typically associated with bat–environment relationships, such as body mass, trophic level, wing morphology, and roost type, were poor predictors of species' responses to environmental variables. However, species' occupancy varied with site temperature (and thus, elevation) in a phylogenetically conserved manner. Based on previous studies, we speculate that physiological traits conserved within certain clades, such as the ability to enter torpor, likely drive these patterns. Our study sheds light on the deterministic drivers of bat occupancy along a heterogeneous environmental gradient and suggests that bat elevational niches are phylogenetically conserved in subtropical Brazil.

## Graphical abstract

We investigated how ecological traits and evolutionary history influence the occupancy of 27 bat species along a 1300 m elevational gradient in subtropical Brazil. While ecological traits poorly predicted species' responses, occupancy varied with temperature in a phylogenetically conserved manner, suggesting that elevational niches in bats may be shaped by phylogenetically conserved and difficult‐to‐measure traits, such as physiological traits.

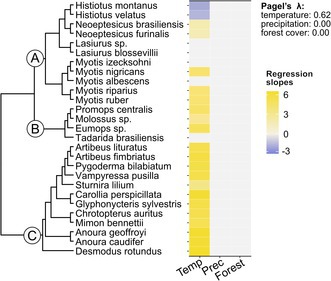

## 1 Introduction

Understanding the processes that shape species distribution and community structure across space and time has long been an active topic in ecology (Vellend 2016), gaining even greater importance with current rates of climate and land‐use change (Mouquet et al. 2015). Recent theoretical syntheses have provided important insights into these dynamics by recognizing the importance of three main processes: niche selection, ecological drift, and dispersal (Leibold and Chase 2018; Vellend 2016). Simulation studies have further enhanced our understanding by showing that the relative effects of each process in a system depend on regional and local environmental factors, such as heterogeneity and connectivity, as well as species' characteristics, including niche breadth and dispersal ability (Khattar et al. 2021; Mouquet and Loreau 2003; Thompson et al. 2020; Vellend 2016).

In steep elevational gradients where environmental conditions change markedly over short geographic distances, niche selection has been consistently identified as the main process shaping species' spatial distribution and community structure (Jarzyna et al. 2021; Khattar et al. 2021; Presley et al. 2012), particularly in taxa with medium to high dispersal ability. If dispersal limitation is minimal, species can colonize all suitable environments, and their distribution becomes a function of the spatio‐temporal variation in fitness and demographic stochasticity (Vellend 2016). This scenario offers a unique opportunity to study species niches, as the effect of dispersal becomes weaker. Not surprisingly, elevational gradients have long been recognized as natural laboratories for ecological research (Malhi et al. 2010; McCain and Grytnes 2010; Tito et al. 2020; Willig and Presley 2016).

Neotropical bats comprise an excellent model group to assess niche‐based dynamics along environmental gradients due to the high taxonomic, ecological, and evolutionary diversity observed in most regions (Bogoni et al. 2021; Stevens 2004). In many South American (sub)tropical forests, these animals often comprise the most abundant and species‐rich mammalian order, with local assemblages composed of up to 100 species (Patterson et al. 1996; Simmons and Voss 1998; Velazco et al. 2021). The maintenance of such high diversity naturally requires strong mechanisms of resource partitioning (Chesson 2000), which are reflected in notable shifts in species niches (Santana et al. 2024). As a consequence, Neotropical bat assemblages are often composed of species exploiting the environments in very different ways (Denzinger and Schnitzler 2013; Kalko et al. 2008).

Previous studies addressing the distribution and diversity of Neotropical bats along elevational gradients have highlighted strong niche selection shaping variation in species distribution (Cisneros et al. 2014; de Carvalho et al. 2019; Mancini et al. 2019; Presley et al. 2012). Highlands often harbor a subset of the species that inhabit low elevations (de Carvalho et al. 2019; Patterson et al. 1996; Presley et al. 2012), and that exhibit ecological traits or evolutionary histories that are either more similar (Cisneros et al. 2014; de Carvalho et al. 2019; Mancini et al. 2019) or more divergent (Cisneros et al. 2014) than expected by chance. There is no consensus, however, on the degree to which these patterns are generalizable across different contexts (e.g., latitudes, species pools, forest types), and identifying these relationships in understudied regions remains a key research focus.

Here, we use a multi‐species modeling approach that explicitly incorporates species' imperfect detection, ecological traits, and phylogeny to investigate the ecological and evolutionary drivers of bat occupancy and species richness along a ~1300 m elevational gradient in the subtropical Brazilian Atlantic Forest. Our study region is characterized by marked environmental heterogeneity, encompassing three forest types, and spans a transition between the bat faunas typical of tropical South America, dominated by phyllostomids, and those of temperate South American ecosystems, dominated by molossids and, especially, vespertilionids (Ramos Pereira and Palmeirim 2013; Stevens 2004).

In addition to mapping species‐environment associations in our study system, we aimed to address a central question: can ecological traits and phylogeny predict bat species occupancy along elevational gradients? We hypothesized that if niche selection shapes species distribution in these systems, as observed in previous studies, then traits and phylogeny should give us clues about the morpho‐ecological attributes selected by environmental conditions at different elevations (Vellend 2016; Webb et al. 2003). Indeed, many traits are suggested to increase or decrease fitness in colder environments (Table 1), and we predicted that these would be important in our system. By testing this prediction, our study helps to elucidate the deterministic drivers of bat species distribution and species richness along subtropical elevational gradients.

**TABLE 1:** Descriptions, ecological relevance, and data sources of ecological traits used to explore trait–environment interactions along an elevational gradient in subtropical Brazil.

Trait	Values	Ecological relevance	Source^a^
Body mass	Species mean value (g)	Influences thermoregulatory efficiency and torpor use (McNab 1969); smaller‐bodied species may cope better with resource scarcity at high elevations	Primary data; 1
Trophic level	Binary variable for trophic level: phytophagous (0) or animalivorous (1)	Phytophagous species may have limited fat storage, which becomes critical in cold environments; plant items and animal prey may vary with elevation in distinct ways	2, 3
Aspect ratio	Species mean value (wingspan^2^ divided by wing area)	High‐aspect‐ratio wings enhance flight efficiency in open habitats (Norberg and Rayner 1987), a trait particularly advantageous in high‐elevation landscapes where forests interface with grasslands	4, 5, 6, 7
Roost type	Binary variable for roosting habitat: foliage (0) or cavity (1)	Cavities buffer against cold; many foliage‐roosting species depend on large leaves, which are less available in frost‐prone forests	8, 9, 10

^a^ 1. Gonçalves et al. (2018); 2. Geiselman and Younger (2020); 3. Carvalho et al. (2008); 4. Norberg and Rayner (1987); 5. Sánchez and Carrizo (2021); 6. Marinello and Bernard (2014); 7. Tavares (2013); 8. Voss et al. (2016); 9. Garbino and Tavares (2018); 10. Barros and Bernard (2023).

## 2 Material and Methods

### 2.1 Study Area

**FIGURE 1:**
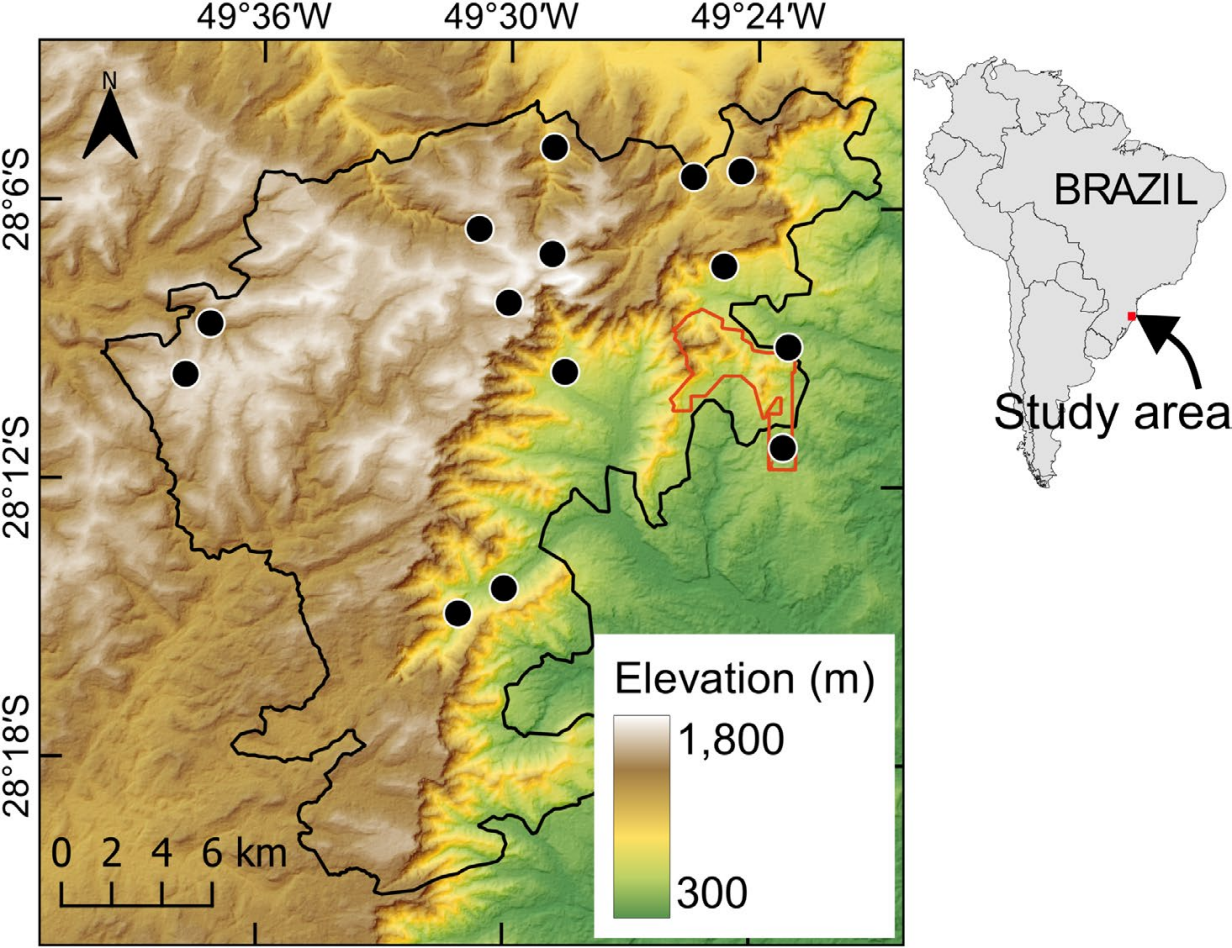
Location of the 14 sites where bats were sampled (solid dots) within the larger São Joaquim National Park (SJNP, black polygon) and the smaller Serra Furada State Park (SFSP, red polygon), in subtropical southern Brazil. The terrain is colour‐coded according to elevation (m.a.s.l.).

Bats were sampled in two contiguous Protected Areas located in the state of Santa Catarina, southern Brazil, amounting to approximately 50,000 ha (Figure 1): São Joaquim National Park (SJNP) and Serra Furada State Park (SFSP). The region is characterized by a montane landscape, with elevations ranging from ~300 to ~1822 m above sea level (m.a.s.l.). The climate is humid subtropical, lacking a dry season (Alvares et al. 2014). During the coldest months (July and August), there are frequent frost events, with mean temperatures ranging from ~7°C to ~14°C in the highland and lowland portions of the study area, respectively. During the hottest months (January and February), average monthly temperatures rise by approximately 8°C. Total precipitation is relatively constant across the study area, exceeding 100 mm/month, but is notably higher in the summer, often surpassing 200 mm/month. The entire region is within the Atlantic Forest domain, but forest types change with elevation (Klein 1978). Tropical rainforests occupy sites below ~900 m.a.s.l. Mixed (Araucaria) Forests emerge above this elevation threshold, where tropical species mix with species typical of subtropical and temperate South American forests, including the widespread dominance of the Brazilian pine ( *Araucaria angustifolia* ). Finally, cloud forests dominate the vegetation upslope of ~1300 m.a.s.l., especially at low‐light, high‐humidity areas. At higher elevations, forests also share the landscape with native grasslands, dominated by shrub species. The study area was subjected to intensive selective logging until the early 20th century. As a result, primary forest remnants are mainly restricted to the steepest areas. The study area is therefore dominated by late‐stage secondary forests, but some small patches of pastures and *Eucalyptus* and *Pinus* forestry also occur.

### 2.2 Selection of Sampling Sites

Fourteen sampling sites were selected to represent the environmental variation across the study area (Figure 1; Table S1). These sites ranged from 450 to 1710 m.a.s.l. and spanned all three main forest types described above. The minimum distance between sites was 1.91 km, and sites were carefully selected to ensure that pairwise geographic distances were not strongly correlated with differences in elevation, thereby minimizing spatial autocorrelation. Forest cover within a 2 km radius around each site exceeded 50% in all cases (mean = 76.6%).

### 2.3 Sampling Procedure

We conducted three sampling seasons: the first one in May 2022, the second in October 2022, and the last in February–March 2023. At each site, we established a 300‐m transect along which we placed seven understorey mist nets (Ecotone 719) and two passive acoustic recording units (AudioMoth v. 1.1.0; hereafter ‘ARUs’). Mist nets were spaced apart by at least 30 m and were positioned along pre‐existing trails, in clearings, and over streams. At each site and season, nets were operated for 5 h from dusk for a single night, resulting in a sampling effort of 900 m^2^•h per site per sampling season, calculated following Straube and Bianconi (2002). ARUs were spaced 200 m apart and remained active for three consecutive full nights (from sunset to sunrise) in each sampling season, recording one 10‐s audio file per minute. This resulted in a sampling effort of 12 recorder•h (or 4320 10‐s audio files) per site per season. Not all sites were sampled using both methods in every season due to unavoidable logistical constraints. In total, we conducted 32 nights of mist net sampling (average of 2.29 per site) and 111 nights of ARU sampling (average of 7.93 per site), resulting in a sampling effort of 28,800 m^2^•h and 444 recorder•h, respectively.

During surveys, all mist nets were checked every 30 min. All captured bats were identified, photographed, measured, and subsequently released. Exceptions included individuals of *Myotis* spp., most of which were collected for more accurate morphological identification and taxonomic harmonization in the laboratory (SISBIO collecting permit No. 80.713‐1 and IMA/SC authorisation No. 02/2022). For ARU sampling, the following recording settings were applied: a sampling rate of 384 kHz, frequency range of 12–192 kHz, and medium gain. Bat calls, defined as any 10‐s audio file containing more than two pulses of a given species, were manually classified using Kaleidoscope Pro v.5.3.9. Nomenclature followed Garbino et al. (2024). Further details on species identification are available in Supporting Information.

### 2.4 Environmental Variables

Elevation of each site was derived from the ASTER Global Digital Elevation Model (GDEM V3; 30 m resolution; earthexplorer.usgs.gov). We used three variables to characterize environmental variation along the elevational gradient: mean annual temperature, total annual precipitation, and forest cover. Temperature and precipitation data were obtained from the Chelsa Climate portal (chelsa‐climate.org; v2.1, ~1 km resolution, reference period: 1980–2010; Karger et al. 2017). Forest cover within 2‐km buffers of each sampling site was calculated from the MapBiomas land‐cover layer (mapbiomas.org; collection 7; 30‐m resolution; Souza et al. 2020). We assessed collinearity among temperature, precipitation, and forest cover using Pearson's correlation coefficient (*r*) and the variance inflation factor (VIF). The values obtained indicated no strong collinearity (*r* = 0.64; VIF = 2.15). Correlations with elevation were −0.99, −0.18, and −0.64 for temperature, precipitation, and forest cover, respectively.

### 2.5 Bat Traits and Phylogeny

We initially considered a set of eight ecological traits linked to bat–environment relationships in previous studies: diet, trophic level, foraging space, foraging mode, roosting preference, aspect ratio, relative wing loading, and body mass. However, some of these traits proved to be highly redundant within our species pool. For instance, open‐ and edge‐space foragers were always aerial insectivores, while phytophagous species were always narrow‐space foragers with low relative wing loading and aspect ratio. We then reduced this set of traits to only four non‐collinear traits, for each of which we erected prior hypotheses regarding their ecological relevance: body mass, trophic level, aspect ratio, and roost type (Table 1). Trait data were obtained from our own primary field data or from specialized literature in a wide range of sources (Barros and Bernard 2023; Carvalho et al. 2008; Garbino and Tavares 2018; Geiselman and Younger 2020; Gonçalves et al. 2018; Marinello and Bernard 2014; Norberg and Rayner 1987; Sánchez and Carrizo 2021; Tavares 2013; Voss et al. 2016).

We used 100 randomly selected posterior samples of the species‐level mammalian tree from Upham et al. (2019) as phylogenetic hypotheses in our analysis. These trees were downloaded from the Vertlife web portal (vertlife.org) using the *rtrees* R package (Li 2023) and processed using the *ape* R package (Paradis and Schliep 2019). For three taxa identified by the acoustic sampling at the genus level (*Eumops* sp., *Lasiurus* sp., and *Molossus* sp.), we considered the phylogenetic position and trait values of the species most likely to occur in our study region, namely *Eumops auripendulus, Lasiurus ega*, and *Molossus fluminensis* (Cherem et al. 2004; Passos et al. 2010). We could not find any information on aspect ratio for four species (*Myotis izecksohni, Eumops* sp., *Histiotus velatus* and *Promops centralis* ) and on roost type for another two species ( *Myotis izecksohni* and *M. ruber* ). These values were then estimated using phylogenetic imputation as implemented in the R package *Rphylopars* (Goolsby et al. 2017).

### 2.6 Phylogenetic Occupancy Models

To explore the role of environmental variables, ecological traits, and evolutionary history on the spatial variation in species occupancy along the elevational gradient, we used Phylogenetic Occupancy Models (POM) that explicitly account for species' imperfect detection and phylogenetic correlation (Frishkoff et al. 2017; Morán‐López et al. 2022). All species were analysed simultaneously, with their parameters (intercepts and coefficients) treated as random effects. Our model treated the true occupancy status of each species (*k*) at each site (*i*), denoted $Z_{k,i}$, as the result of a Bernoulli trial with probability $\psi_{i,k}$. Then, spatial variation in $\psi_{i,k}$ was modelled as a logit‐linear function of mean temperature (Temp), total precipitation (Prec) and forest cover (Forest):

$$Z_{k,i}\sim \text{Bernoulli}\left(\psi_{i,k}\right)$$

$$\text{logit}\left(\psi_{i,k}\right)=\alpha_{k}+\beta_{1,k}*{\text{Temp}}_{i}+\beta_{2,k}*{\text{Prec}}_{i}+\beta_{3,k}*{\text{Forest}}_{i}$$

Where $\alpha_{k}$ is the species‐specific intercept and $\beta_{1,k}$, $\beta_{2,k}$, and $\beta_{3,k}$ are species‐specific coefficients for environmental variables. Following the formulation of Frishkoff et al. (2017), our POM incorporates phylogenetic correlation in species responses to environmental variables by drawn species coefficients from multivariate normal distributions with variance–covariance structure incorporating phylogenetic information scaled by Pagel's *λ* parameter (Pagel 1999). Pagel's *λ* is estimated during model fitting and ranges from 0 to 1, with values close to 1 indicating fully phylogenetically conserved parameters, and values near 0 indicating parameters that are random with respect to phylogeny (Revell 2010).

To test the effect of ecological traits on species responses to environmental variables, we reran the model described above to include ecological traits as linear predictors of species' regression coefficients (Frishkoff et al. 2017; Ovaskainen and Abrego 2020). Thus, we could obtain trait–environment interaction coefficients while controlling for residual phylogenetic signal, which results in more precise estimates and adequate Type 1 error rates (Li and Ives 2017). We fitted models both without (model 1) and with (model 2) traits and compared results in terms of Pagel's *λ*. Additionally, we also calculated Pagel's *λ* in all traits individually using the *phytools* R package (Revell 2024).

We finalised our POM formulation by including a hierarchical sub‐model to account for imperfect detection. This is made by treating the field data of each species at each site, denoted $Y_{i,k}$, as a product of their latent occupancy state $Z_{k,i}$ and detection probabilities ($p_{k}$). We included a first detection likelihood for mist‐netting data and, for 10 species that were either additionally or exclusively detected via ARUs, we incorporated a second detection likelihood, resulting in a joint likelihood model (see Miller et al. 2019). In both cases, we used a binomial formulation that links the number of sampling seasons in which a species was detected at a given site to the number of sampling seasons conducted there ($N_{i}$) and $p_{k}$. As with other parameters, $p_{k}$ was treated as a random effect, with mean and variance estimated at the community‐level (Kéry and Royle 2015).

$$Y_{i,k}\sim \text{Binomial}\left(N_{i},p_{i,k}\right)$$

$$p_{i,k}=p_{k}*Z_{k,i}$$

Our models were implemented in a Bayesian framework using the R package *nimble* v1.1.0 (de Valpine et al. 2017). We ran three Markov Chain Monte Carlo (MCMC) chains, each with 800,000 iterations, a thinning interval of 200, and discarded 600,000 iterations as burn‐in. We assessed model convergence using the Gelman–Rubin R‐hat diagnostic (Brooks and Gelman 1998), ensuring that values were less than 1.1. Prior to analysis, environmental variables and continuous ecological traits were rescaled (mean = 0, SD = 1). We used vague priors for all model parameters except for Pagel's *λ*, for which we applied a Bayes factor prior specification: 50% of the prior mass was fixed at 0, and the remaining 50% were allowed to vary uniformly between 0 and 1. This approach helps to avoid spurious detection of phylogenetic signal that is not supported by the data (Frishkoff et al. 2017; Ovaskainen and Abrego 2020). Bayes factor values greater than 3, measured by the ratio of values greater than zero in the posterior distribution, were interpreted as indicating well‐supported evidence of phylogenetic signal. To integrate phylogenetic uncertainty over 100 available phylogenies during the model fit, we followed Villemereuil et al. (2012), randomly selecting a phylogeny in each MCMC iteration. After model fitting, we extracted the posterior mean and 95% Bayesian credible intervals (BCIs) for parameters of interest.

### 2.7 Species Richness Estimates

To explore how species' responses to environmental variables impact bat species richness along the elevational gradient, we calculated site‐level species richness. We computed both observed richness, based on the raw field data, and the estimated richness, derived from posterior means of species‐specific occupancy estimates generated by our POM, where the distribution of each species was modeled individually while accounting for imperfect detection. We then used Poisson GLMs with a log‐link to assess the relationships between these metrics and (i) elevation (linear and quadratic terms); and (ii) temperature, precipitation, and forest cover.

## 3 Results

**FIGURE 2:**
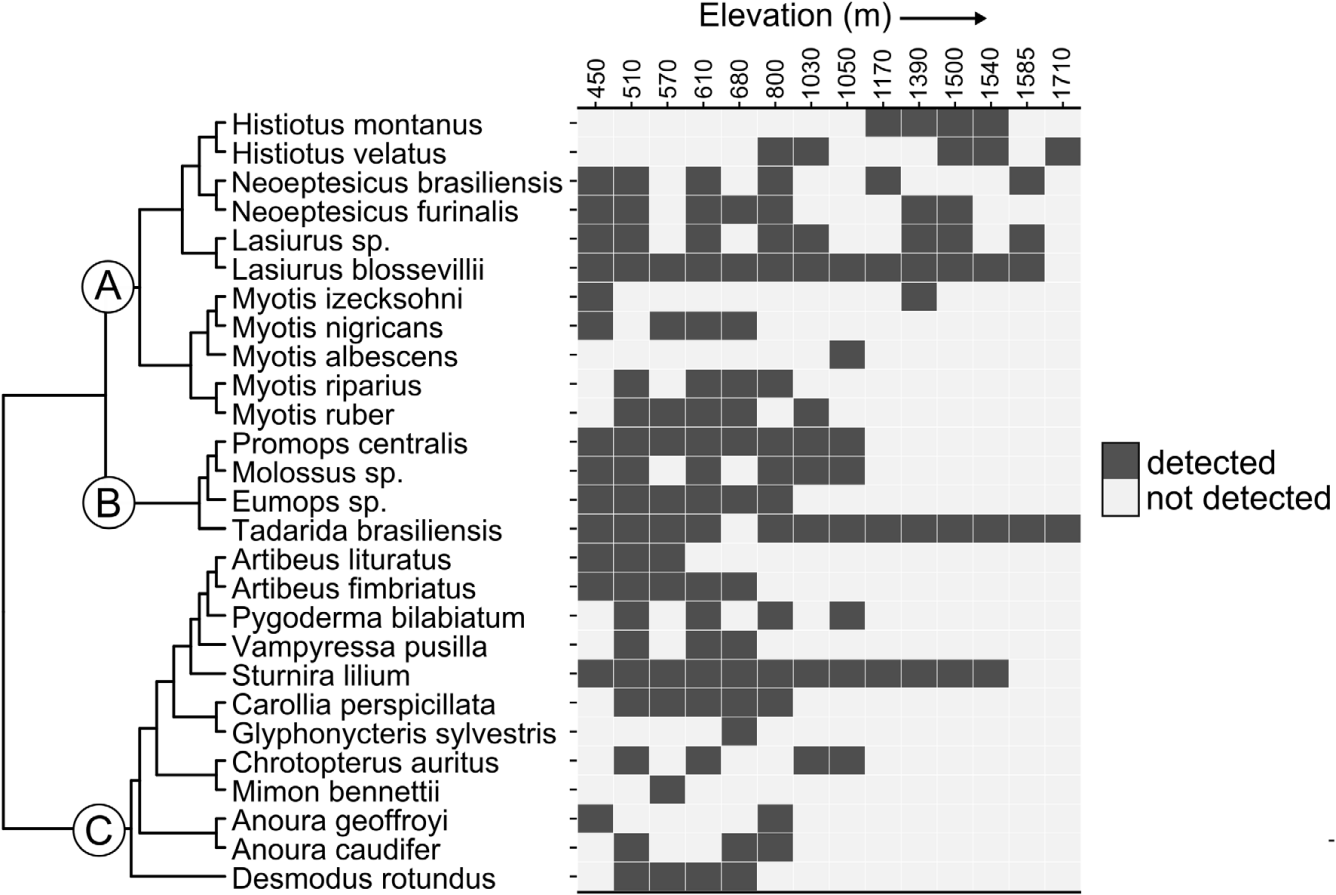
Spatial variation in bat detections along a steep elevational gradient in subtropical Brazil. Sites are ordered by elevation (*X*‐axis), ranging from 450 m (left) to 1710 m (right) above sea level. The phylogenetic tree is based on a single posterior sample from Upham et al. (2019), with emphasis on the families Vespertilionidae (A), Molossidae (B), and Phyllostomidae (C).

We recorded 27 bat species representing three families (Phyllostomidae, Vespertilionidae, and Molossidae; Figure 2). Mist net sampling resulted in the capture of 338 individuals representing 22 species. Acoustic sampling resulted in 1937 bat‐passes representing 10 taxa, five of which (*Neoeptesicus brasiliensis, N. furinalis*, *Histiotus montanus* , *H. velatus* and *Lasiurus blossevillii* ) were also recorded in mist nets. Most species were restricted to low elevations (< 1050 m), while *Histiotus montanus* and *H. velatus* were more frequent at high elevations, and *Tadarida brasiliensis, Sturnira lilium*, and some vespertilionids such as *Lasiurus* spp. and *Neoeptesicus* spp. were elevation generalists, occurring across a wide range of elevations (Figure 2).

Some clades were more frequently detected at high elevations than others (Figure 2). For instance, although more than half of the 12 vespertilionid species had at least one record above 1050 m, *Sturnira lilium* and *Tadarida brasiliensis* were the only species recorded at those elevations in the families Phyllostomidae and Molossidae, respectively.

**FIGURE 3:**
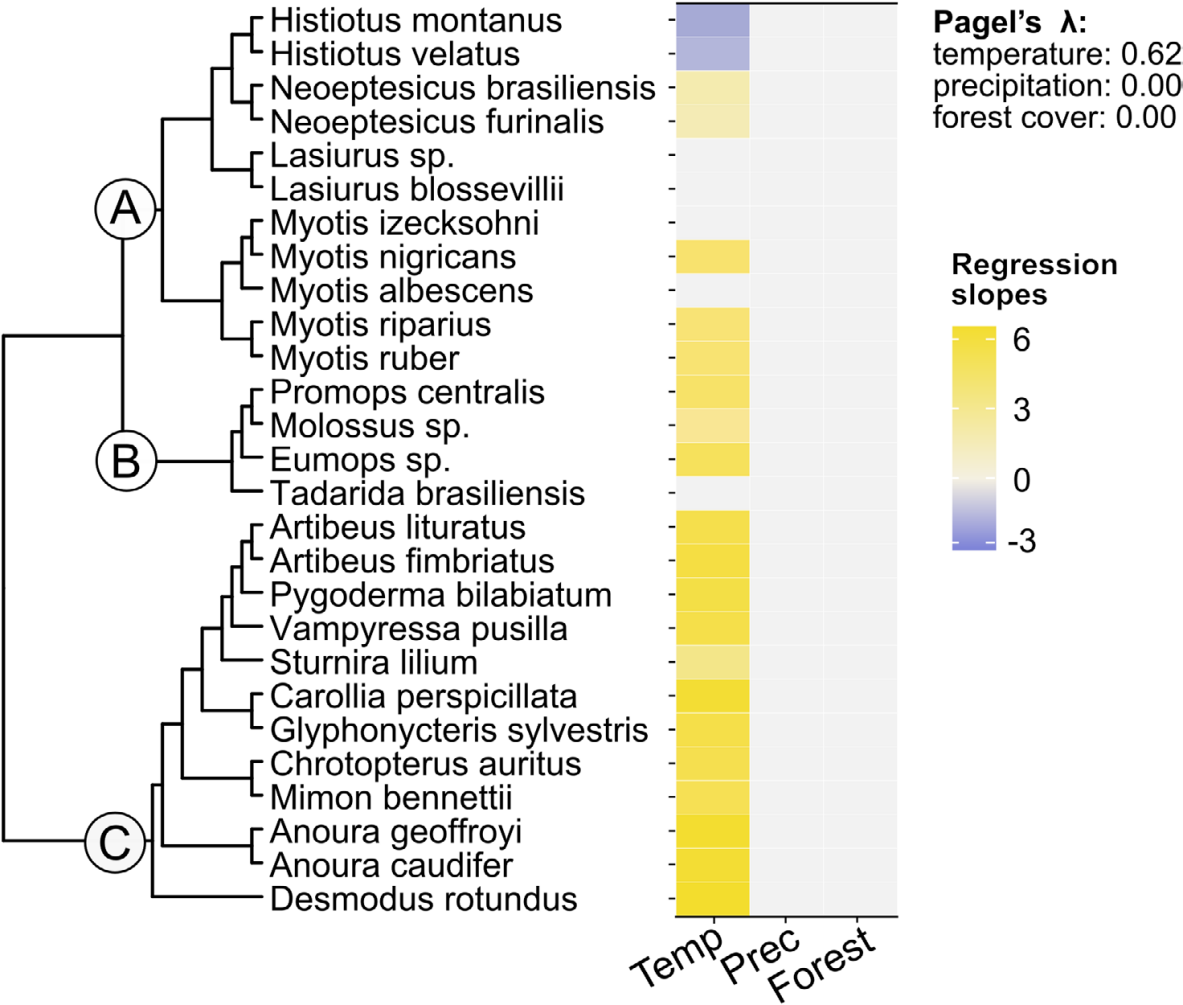
Phylogenetic signal in bat species responses to environmental variables along a steep elevational gradient in subtropical southern Brazil. Values represent posterior means of species‐specific regression coefficients in a Bayesian phylogenetic occupancy model, with positive and negative values shown in yellow and blue, respectively. Coefficients with 95% credible intervals overlapping zero were fixed at zero for illustration purposes. The phylogenetic tree on the left is based on a single posterior sample from Upham et al. (2019), with emphasis on the families Vespertilionidae (A), Molossidae (B), and Phyllostomidae (C).

Our first phylogenetic occupancy model revealed that the occupancy of most species is strongly linked to site temperature (Figure 3), with moderate but well‐supported phylogenetic signal in these relationships (Pagel's *λ* = 0.62 [95% BCI: 0.17–0.96], Bayes factor = 10.6). Most species exhibited positive responses to this variable (regression coefficients greater than zero), except for *Histiotus montanus* and *H. velatus* , which showed well‐supported negative responses, indicating that their occupancy probability increases at colder sites. In contrast, there was no evidence of an association between precipitation and forest cover with species occupancy, as BCI values overlapped zero in all regression coefficients. Species responses to both these variables exhibited no phylogenetic signal (Bayes factor = 0.9 and 1.0 for precipitation and forest cover, respectively).

**FIGURE 4:**
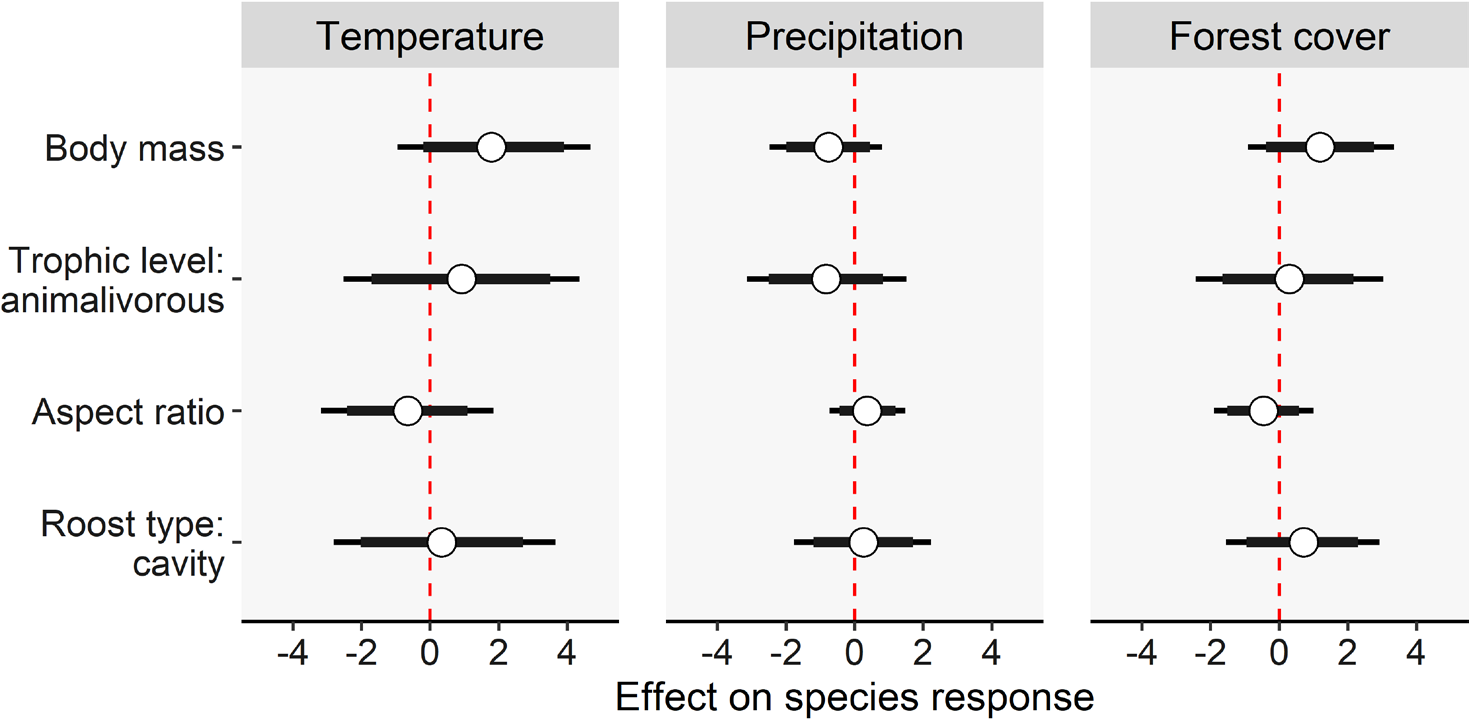
Effects of trait–environment interactions on bat species occupancy along a steep elevational gradient in subtropical Brazil. Coefficients were derived from a Bayesian phylogenetic occupancy model. Open circles indicate posterior means, with thick and thin bars representing 85% and 95% credible intervals, respectively. A positive coefficient indicates that species with higher values of a given trait (e.g., body mass) exhibit higher occupancy probabilities at sites with higher values for the corresponding variable (e.g., temperature).

Except for roost type (*λ* = 0.00), there was phylogenetic signal in all traits considered here (body mass: *λ* = 0.99; trophic level: *λ* = 1.00; aspect ratio: *λ* = 0.92). However, our second phylogenetic occupancy model including trait‐environment relationships while accounting for phylogeny failed to find any evidence of an association between species responses to environmental variables and ecological traits, as 95% BCIs overlapped zero in all cases (Figure 4). Furthermore, even after accounting for the effect of traits, there was residual phylogenetic signal in species responses to temperature (Pagel's *λ* = 0.62 [95% BCI: 0.11–0.97], Bayes factor = 4.5), but not to precipitation (Bayes factor = 1.2) or forest cover (Bayes factor = 1.1). More details on POM estimates are available in Supporting Information.

**FIGURE 5:**
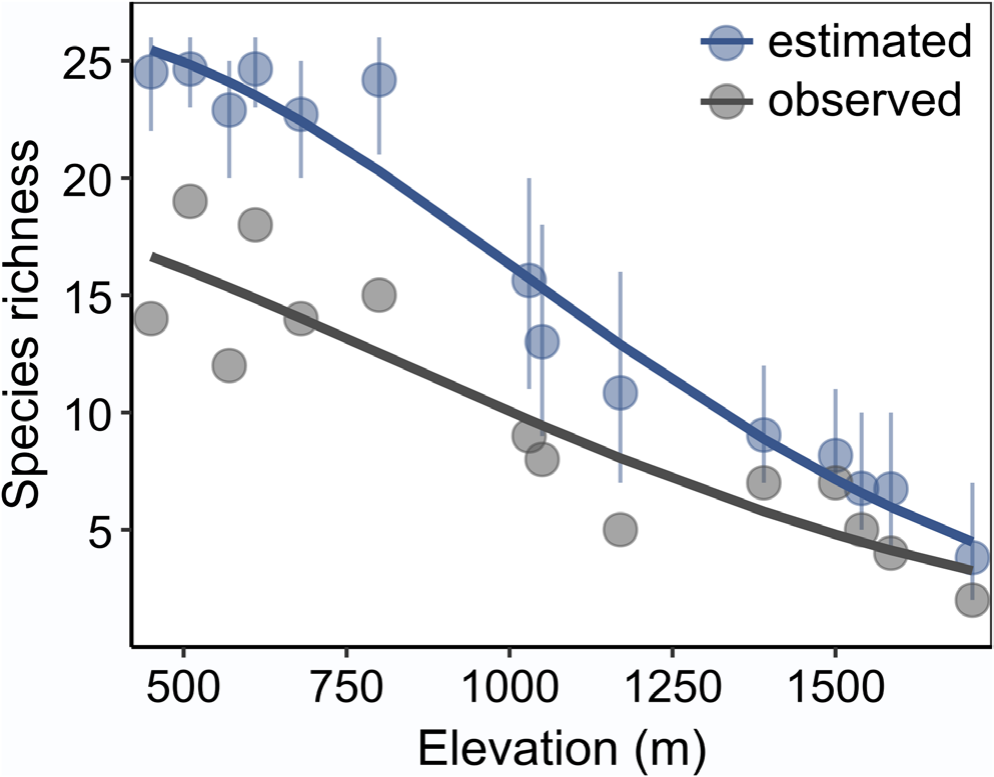
Relationship between observed (grey) and detectability‐corrected (blue) bat species richness and elevation (m.a.s.l.) along a steep elevational gradient (450–1710 m) in subtropical southern Brazil. Estimated richness (blue) is represented by posterior means (dots) and 95% credible intervals (error bars). Solid lines depict best‐fit regression curves from Poisson generalised linear models.

Observed site‐level species richness ranged from 2 to 19 species and declined linearly with elevation (Table 2; Figure 5). Estimated site‐level species richness derived from our phylogenetic occupancy model ranged from 4 (95% BCI 2–6) to 25 (95% BCI 23–26) species and also declined linearly with elevation (Table 2; Figure 5). When modelled as a function of environmental variables, both measures of species richness were positively associated with temperature but not with precipitation or forest cover (Table 2), which is consistent with the results from species‐specific analyses.

**TABLE 2:** Summary of Poisson generalized linear models (GLMs) examining relationships between observed and estimated species richness and environmental variables.

Term	Estimate	*Z*‐value	*p*
*Observed richness ~ elevation*			
Intercept	2.26	15.03	< 0.001
Elevation	−0.57	−5.34	< 0.001
Elevation^2^	−0.12	−0.85	0.398
*Estimated richness ~ elevation*			
Intercept	2.77	23.44	< 0.001
Elevation	−0.58	−6.76	< 0.001
Elevation^2^	−0.17	−1.50	0.133
*Observed richness ~ environmental variables*			
Intercept	2.16	22.25	< 0.001
Temperature	0.51	3.98	< 0.001
Precipitation	0.06	0.56	0.578
Forest cover	0.05	0.37	0.714
*Estimated richness ~ environmental variables*			
Intercept	2.63	34.24	< 0.001
Temperature	0.54	5.37	< 0.001
Precipitation	0.09	1.08	0.281
Forest cover	−0.02	−0.20	0.838

## 4 Discussion

By employing a unifying framework that integrated mist net and acoustic recorder data, environmental variables, ecological traits, and phylogeny, our study sheds light on the deterministic drivers of bat distribution and species richness along a steep elevational gradient in subtropical southern Brazil. Most species were more frequent at low‐elevation sites, where mean annual and winter temperatures are higher, indicating that temperature—and/or its ecological correlates—acts as a strong environmental filter for these bats. Exceptions included *Histiotus montanus* and *H. velatus* , two congener vespertilionids that were restricted to high‐elevation sites. Contrary to expectations, ecological traits typically associated with bat–environment relationships showed limited explanatory power, with occupancy patterns instead strongly structured by phylogenetic relatedness. These results suggest that phylogeny captures latent ecological variation linked to traits that are inherently challenging to quantify directly (Cadotte et al. 2013), such as physiological ability to enter torpor, which likely underpins bat species response to environmental variation along this subtropical elevational gradient.

### 4.1 Environmental Drivers of Bat Distribution Along Elevational Gradients

We evaluated site temperature, precipitation, and forest cover as potential environmental drivers of bat distribution and local species richness along the studied gradient, but our results suggest that only ambient temperature plays an important role. It exerted well‐supported effects on the occupancy of 22 of the 27 studied species and was the sole environmental variable explaining variation in species richness. Indeed, temperature has been identified as a key driver of anuran species richness and assemblage structure in the subtropical Atlantic Forest (Bassetto et al. 2024; Carvalho‐Rocha et al. 2021), underscoring its broad importance for vertebrate communities in the region. This also aligns with broader evidence that temperature seasonality shapes bat species richness gradients across the entire Atlantic Forest (Stevens 2013), highlighting the role of extreme temperatures in limiting bat occupancy even at larger spatial scales.

In contrast, precipitation and forest cover did not influence the occupancy of any of the species examined here. Precipitation, alongside temperature, has been proposed as a key variable explaining elevational gradients in bat species richness worldwide (McCain 2007). Specifically, McCain's (2007) climate model predicts that bat richness peaks in portions of the gradient with higher temperature and water availability. However, our study area is relatively humid throughout its entire extent, with annual precipitation exceeding 1890 mm at all sites. These high precipitation rates likely provide sufficient ecological conditions and productivity required for the occurrence of the study species, explaining the lack of observed associations. The same applies to forest cover, which exceeds 50% at all sites in our largely protected study area—or well above the 30% threshold suggested as necessary to maintain the integrity of Atlantic Forest vertebrate communities (Banks‐Leite et al. 2014). In elevational gradients where these variables vary more markedly, both, alongside temperature, may influence bat species richness and distribution to some degree (McCain 2007). Furthermore, other variables for which we lacked locally derived data—such as vertical forest structure, secondary productivity, and roost availability—may also be relevant in certain contexts (de Carvalho et al. 2019; Presley et al. 2012) and warrant consideration in future studies.

### 4.2 Ecological Traits and Phylogeny as Predictors of Species Distribution

Contrary to our predictions and previous studies (e.g., Cisneros et al. 2014; de Carvalho et al. 2019; Mancini et al. 2019), ecological traits typically associated with bat–environment relationships failed to predict species responses to environmental variables. However, responses to temperature exhibited a marked phylogenetic signal. Highland assemblages were dominated by Vespertilionids, while Phyllostomids and Molossids were each represented by only one species. This pattern mirrors findings in other Brazilian Atlantic Forest montane regions (Cherem and Althoff 2015; Mancini et al. 2019; Martins et al. 2015; Miranda et al. 2019), and aligns with broader latitudinal trends in South America, where vespertilionids dominate temperate climates (Ramos Pereira and Palmeirim 2013; Stevens 2004). Since this is a clade for which torpor and hibernation are frequently observed during harsh conditions (Geiser and Stawski 2011; McNab 1969; Odon et al. 2023), our results are in line with predictions that highland portions of (sub)tropical elevational gradients are primarily comprised of niche‐selected bat species from clades physiologically capable of entering torpor during cold seasons (Cisneros et al. 2014; Graham 1983). Species from clades of tropical origin (e.g., Phyllostomidae), which evolved in thermally stable ecosystems, likely lack the physiological mechanisms to persist at colder sites, at least during winter months (Mello et al. 2008).

Fine scale dietary variation emerges as another trait that is challenging to quantify directly but may help explain variation in bat species responses to temperature. For instance, inherently tropical plants such as *Cecropia* spp., *Ficus* spp., *Piper* spp., and *Vriesea* spp., which are important fruit and nectar sources for phytophagous bats such as *Artibeus* spp., *Carollia perspicillata, Vampyressa pusilla*, and *Anoura* spp. (Geiselman and Younger 2020; Passos et al. 2003; Sazima et al. 1999), are much more abundant or even entirely restricted to lowland forests in our study area (de Gasper et al. 2013; Lingner et al. 2015; personal observation), where winters are mild and average annual temperatures are higher. This helps to explain why these bat species are restricted to low elevations. In contrast, *Solanum* spp., the primary food source for *Sturnira lilium* (Mello et al. 2008), is ubiquitous along elevational gradients of the subtropical Atlantic Forest (de Gasper et al. 2013; Mello et al. 2008), likely enabling a broad elevational range for this phyllostomid bat. Additionally, similar dietary constraints may apply to insectivorous bats, but we have no information available on the degree to which invertebrate prey fluctuates with elevation in our study area.

The absence of an association between measured traits and environmental variables was unexpected, given prior evidence linking traits such as body size, diet, and roost type to the elevational distribution of bats in tropical South America (Cisneros et al. 2014; de Carvalho et al. 2019; Mancini et al. 2019). For instance, bats were smaller‐bodied than expected by chance in highland assemblages along an Andean elevational gradient in Peru (Cisneros et al. 2014), whereas roost type was an important trait explaining the response of phyllostomid bats to elevation in the Atlantic Forest of southeastern Brazil (de Carvalho et al. 2019). While it is possible that these relationships simply do not apply to our subtropical study area, or that they are obscured by the greater importance of physiological traits in this system, discrepancies may reflect methodological differences. First, our approach integrating all traits and phylogeny in a single model reduces type 1 errors compared to single‐trait approaches used in some of these studies (Li and Ives 2017; Peres‐Neto et al. 2017). Second, our species pool (27 species) was relatively small, suggesting that the set of species studied may be insufficient to detect weaker patterns observed previously. This is counterbalanced, however, by the strong phylogenetic signal observed, indicating that at least one unmeasured phylogenetically conserved ecological trait had detectable effects in our data (Cadotte et al. 2013).

### 4.3 Implications for Bat Conservation

Rising global temperatures highlight the urgent need to assess species' vulnerability to climate change (Pacifici et al. 2015). In our study, temperature emerged as a critical driver of bat distribution and species richness, suggesting future warming could profoundly reshape elevational patterns. The high‐elevation sites in our study area—among the coldest regions in Brazil—are projected to experience significant warming. Cold‐adapted bats, such as *Histiotus montanus* and *H. velatus* , may face heightened extinction risks due to the progressive loss of climatically suitable habitats. Indeed, these vespertilionid bats appear to be restricted to cold, high‐elevation or high‐latitude environments throughout their entire geographic range (da Silva et al. 2021; de Carvalho et al. 2013; Ossa et al. 2015), suggesting that they may be at risk even across broader spatial scales. Alarmingly, to the best of our knowledge, no monitoring programs target these species in the subtropical Atlantic Forest, and they remain absent from most regional Red Lists. This reflects a broader pattern of neglected bat conservation needs in Brazil and elsewhere in South America (Aguiar and Ramos Pereira 2019). Proactive measures, including habitat connectivity initiatives and identification and protection of climate refugia, are essential to safeguard these ecologically specialized species (Morelli et al. 2016; Nuñez et al. 2013).

## Author Contributions

**Carlos Henrique Russi:** conceptualization (equal), data curation (lead), formal analysis (lead), methodology (equal), project administration (lead), writing – original draft (lead), writing – review and editing (equal). **Selvino Neckel‐Oliveira:** conceptualization (equal), project administration (equal), supervision (equal), writing – original draft (equal), writing – review and editing (equal). **Vítor Carvalho‐Rocha:** formal analysis (supporting), methodology (supporting), software (supporting), writing – review and editing (equal). **Carlos A. Peres:** conceptualization (equal), methodology (equal), project administration (equal), writing – original draft (equal), writing – review and editing (equal).

## Ethics Statement

This study was authorized and registered under licenses from Instituto Chico Mendes de Conservação da Biodiversidade—ICMBio (SISBIO collecting permit No. 80.713–1) and from Instituto do Meio Ambiente de Santa Catarina (authorisation No. 02/2022).

## Conflicts of Interest

The authors declare no conflicts of interest.

## Supporting information


**Data S1:** ece371912‐sup‐0001‐Supinfo.docx.

## Data Availability

Data and code required to reproduce the main analyses are available in the Figshare repository: https://figshare.com/s/5fee0bc1b6510800c9fa.
