# Supplementary material for: Phylogenetic Conservatism and Ambient Temperature Shape Spatial Variation in Bat Occupancy and Species Richness Along a Subtropical Elevational Gradient
Source: Ecol Evol. 2025 Sep 25;15(10):e71912. doi: 10.1002/ece3.71912 (PMC12461109; doi:10.1002/ece3.71912)
Supplement: Supplementary file 1 — Data S1: ece371912‐sup‐0001‐Supinfo.docx. [file ECE3-15-e71912-s001.docx]

supporting informaTion

# Phylogenetic conservatism and ambient temperature shape spatial variation in bat occupancy and species richness along a subtropical elevational gradient

**Sampling site description**

We selected 14 sites to represent the environmental heterogeneity present within our study area (Table S1). These sites ranged from 450 to 1,710 m above sea level, spanning almost the entire elevation gradient of the Serra do Mar mountains in Santa Catarina and including the three main local forest types: cloud Forest, Araucaria Forest and lowland rainforest.

Table S1. *Identification numbers, site codes, geographical coordinates, elevation (m a.s.l.), and associated forest type for the 14 surveyed sites across a steep elevational gradient in subtropical southern Brazil*.

| **ID** | **Site code name** | **Longitude** | **Latitude** | **Elevation (m a.s.l.)** | **Forest type** |
| --- | --- | --- | --- | --- | --- |
|  |  |  |  |  |  |
| 1 | Nebular da Igreja | -49.483 | -28.118 | 1,710 | Cloud forest |
| 2 | Nebular Sta. Bárbara | -49.631 | -28.162 | 1,585 | Cloud forest |
| 3 | Grota Rio Pelotas | -49.500 | -28.135 | 1,540 | Cloud forest |
| 4 | Godinho | -49.512 | -28.109 | 1,500 | Cloud forest/Araucaria |
| 5 | Cascatinha Sta. Bárbara | -49.621 | -28.144 | 1,390 | Cloud forest/Araucaria |
| 6 | Morro comprido | -49.412 | -28.084 | 1,170 | Cloud forest/Araucaria |
| 7 | Fazenda Cachimbo | -49.482 | -28.079 | 1,050 | Araucaria |
| 8 | Rio do Bispo | -49.425 | -28.091 | 1,030 | Araucaria |
| 9 | Pirâmides | -49.413 | -28.121 | 800 | Rainforest |
| 10 | Gruta | -49.519 | -28.247 | 680 | Rainforest |
| 11 | Fazenda | -49.477 | -28.160 | 610 | Rainforest |
| 12 | Sede norte PAESF | -49.387 | -28.150 | 570 | Rainforest |
| 13 | Casinha | -49.500 | -28.237 | 510 | Rainforest |
| 14 | Sede sul PAESF | -49.388 | -28.186 | 450 | Rainforest |

The study region's climate is subtropical humid without a dry season (Alvares et al., 2014). At our high-elevation study sites (>800 m a.s.l.), frost events are common during winter, and mean temperatures during the coldest month (July) can reach close to 7°C (Figure S1). On the other hand, winter is milder at low elevation sites, with mean temperatures close to 14°C (Figure S1). Compared to winter, mean temperatures during summer (from December to February) can increase by up to 8°C. Monthly total precipitation is similar throughout the entire study area, but is noticeably higher in the summer, when it exceeds 200 mm/month (Figure S1).


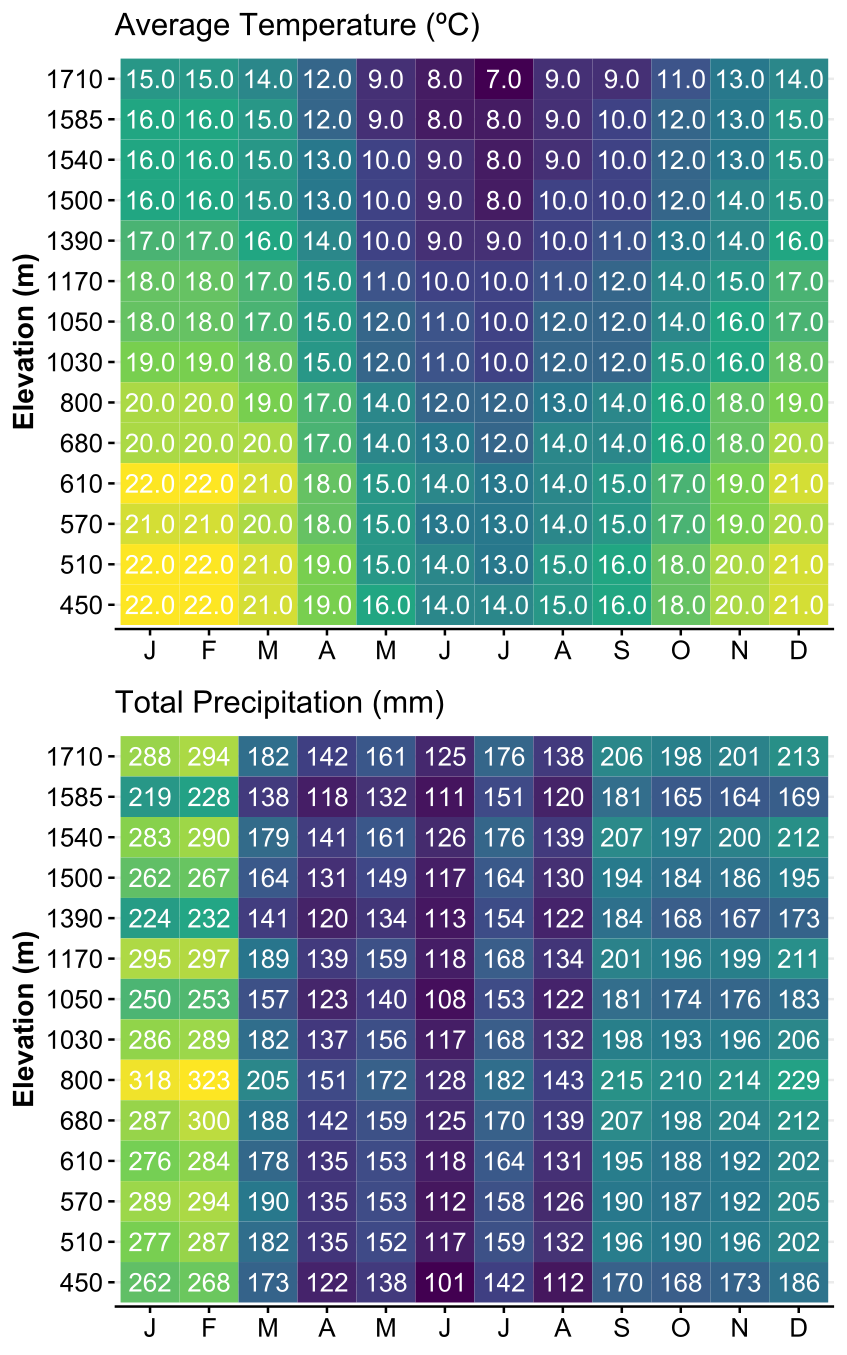


Figure S1. Monthly averaged temperature (°C; top) and total precipitation (mm; bottom) for the 14 sites surveyed across a steep elevational gradient in subtropical southern Brazil. Sites are represented by rows, months by columns, and colour gradients indicate variation in measured values. Letters on the x-axis correspond to months sequentially from January to December.

**Species identification**

We followed the nomenclature recommended by Garbino et al. (2024). Captured specimens were identified in the field. Exceptions were individuals of *Myotis* spp., almost all of which were collected for laboratory identification (SISBIO Authorization No. 80.713-1 and IMA/SC Research Authorization No. 02/2022) and then deposited into the Zoological Collection of the Regional University of Blumenau (CZFURB). Identification followed available guides for South American bats (Díaz et al., 2021; Novaes et al., 2022; Reis et al., 2007).

Recorded calls were manually identified in Kaleidoscope v. 4.5.6, based on previously described characteristics (Arias-Aguilar et al., 2018; Falcão et al., 2021; Jung et al., 2014; López-Baucells, 2018), in addition to our personal bat call library. Specifically, we used six call parameters to identify bat species: structure, number of distinct pulses, bandwidth, duration, minimum frequency and frequency of maximum energy. Calls that could not be attributed to a single species were grouped at genus level if the candidate species was not mist-netted (i.e. *Eumops* sp., *Lasiurus* sp. and *Molossus* sp.), or discarded if they were (i.e. *Myotis* spp.). Although the amount of data on the occurrence of *Myotis* spp. obtained from acoustic recorders was considerably greater than that obtained from mist nets, we chose to use only the mist net data because it was not possible to distinguish the calls of each of the five species of the genus occurring in the region. Additionally, some of the candidate species do not even have their calls described (e.g. *M. izecksohni*). We were then able to identify ten species from acoustic recordings. Their observed acoustic parameters are given in Table S2.

Table S2. Bat species and associated acoustic parameters derived from calls recorded across a steep elevational gradient in subtropical southern Brazil. Continuous call parameters are presented as mean ± standard deviation for selected recordings. Key references used for species identification are included. Acronyms: Fmin, minimum frequency (kHz); FME, frequency of maximum energy (kHz); Bw, bandwidth (kHz); Dur, call duration (ms).

| **Species** | **Structure** | **Pulse** | **Fmin**  **(kHz)** | **FME**  **(kHz)** | **Bw**  **(kHz)** | **Dur**  **(ms)** | **References** | **Comments** |
| --- | --- | --- | --- | --- | --- | --- | --- | --- |
| *Neoeptesicus furinalis* | FMd-QCF | single | 37.59  ±1.09 | 40.39  ±1.11 | 27.19  ±4.94 | 4.26  ±0.99 | Arias-Aguilar et al. (2018) | Cross-referencing information between captures with mist nets and acoustic recordings supports identification |
| *Neoeptesicus brasiliensis* | FMd-QCF | single | 31.26  ±1.60 | 34.36  ±1.27 | 25.20  ±3.77 | 7.95  ±1.07 | Arias-Aguilar et al. (2018) | Cross-referencing information between captures with mist nets and acoustic recordings supports identification |
| *Histiotus montanus* | FMd-QCF | single | 26.61  ±1.58 | 31.40  ±1.40 | 24.21  ±4.76 | 4.29  ±0.41 | Arias-Aguilar et al. (2018) | Acoustic parameters from calls recorded during the hand-release of individuals captured using mist nets supports identification |
| *Histiotus velatus* | FMd-QCF | single | 15.95  ±0.89 | 18.03  ±1.39 | 10.72  ±2.53 | 4.48  ±1.25 | Arias-Aguilar et al. (2018) | Acoustic parameters from calls recorded during the hand-release of individuals captured using mist nets supports identification |
| *Lasiurus blossevillii* | FMd-QCF | single | 43.54  ±1.26 | 44.75  ±2.76 | 20.00  ±3.96 | 6.05  ± 1.52 | Arias-Aguilar et al. (2018); Falcão et al. (2021) | Cross-referencing information between captures with mist nets and acoustic recordings supports identification |
| *Lasiurus* sp. | FMd-QCF | single | 28.85  ±0.88 | 31.54  ±1.85 | 14.35  ±6.46 | 9.02  ±1.48 | Arias-Aguilar et al. (2018); López-Baucells (2018) | Probably *Lasiurus ega*, but *Lasiurus egregius* has similar parameters and has already been recorded near the study area. Therefore, we opted for a more conservative approach and considered all these calls as "*Lasiurus* sp." |
| *Molossus* sp. | QCFd | low | 25.12  ±1.25 | 26.48  ±1.17 | 2.51  ±1.15 | 11.69  ±0.95 | Arias-Aguilar et al. (2018); Jung et al. (2014); López-Baucells (2018) | Probably *Molossus fluminensis*, which was previously recognized as *M. rufus* (Loureiro et al., 2020)*.* However, *M. currentium* has similar call parameters and has already been recorded near the study area. Therefore, we opted for a more conservative approach and considered all these calls as "*Molossus* sp." |
|  |  | middle | 30.18  ±1.34 | 31.08  ±1.50 | 2.38  ±1.19 | 11.155  ±2.03 |  |  |
|  |  | high | 32.50  ±1.96 | 33.20  ±1.84 | 10.02  ±2.34 | 15.41  ±1.31 |  |  |
| *Eumops* sp. | QCFd | low | 17.08  ±1.56 | 20.38  ±1.69 | 10.48  ±1.09 | 14.25  ±2.30 | Arias-Aguilar et al. (2018); Falcão et al. (2021); Jung et al. (2014) | Probably *Eumops auripendulus*, but *E. bonarienses* is also expected to occur in the study area. Because we could not find any information concerning the differences between these species call parameters, we opted for a more conservative approach and considered all these calls as "*Eumops* sp." |
|  |  | high | 23.25  ±1.01 | 24.45  ±1.27 | 6.15  ±1.14 | 10.02  ±2.01 |  |  |
| *Promops centralis* | FMu-QCF | low | 28.91  ±1.17 | 30.23  ±1.22 | 2.26  ±1.06 | 10.23  ±1.59 | Arias-Aguilar et al. (2018); Hintze et al. (2020); Jung et al. (2014) | We mainly recorded “type I” calls *sensu* Hintze et al. (2020), occasionally alternating with FMd-QCF calls. |
| *Tadarida brasiliensis* | QCFd | single | 23.24  ±1.32 | 24.63  ±1.46 | 2.20  ±0.96 | 13.64  ±2.54 | Arias-Aguilar et al. (2018); Jung et al. (2014) | *Nyctinomops laticaudatus* has similar call parameters (Jung et al., 2014), but previous studies (Bôlla et al., 2017) and our personal observation suggest that this species does not occur in our study region, while *T. brasiliensis* is quite common. |

**Phylogenetic occupancy models**

We employed a Bayesian Phylogenetic Occupancy Model (POM) to assess species responses to environmental variables while accounting for phylogenetic correlations among species parameters and imperfect detection. POMs extend the single-species occupancy framework of Mackenzie et al., (2002) into a hierarchical structure, where occupancy and detection parameters for all species are estimated simultaneously, treated as random effects drawn from community-level distributions. For this study, we further adapted POMs to include two distinct detection submodels: one for mist net data (all species) and another for Acoustic Recording Unit (ARU) data, applied to the 10 species detected either exclusively or additionally by ARUs. This adaptation renders our POM a joint likelihood model (Miller et al., 2019). Details on model implementation and occupancy-related results are presented in the main text; here, we focus on the detection component.

Detection probabilities in a single mist net survey were low (mean = 0.13 [0.08–0.20]) and varied considerably between species (SD = 1.48 [logit scale]). Vespertilionids and especially molossids exhibited near-zero detection probabilities, whereas *Sturnira lilium*—the most abundant phyllostomid in our study area—had its detection probability estimated at 0.83. Conversely, detection probabilities in a single ARU survey were higher (mean = 0.49 [0.35–0.62]) and less variable across species (SD = 0.84 [logit scale]), with similar values observed for both vespertilionids and molossids. Species-specific detection probabilities are illustrated in Figure S2.


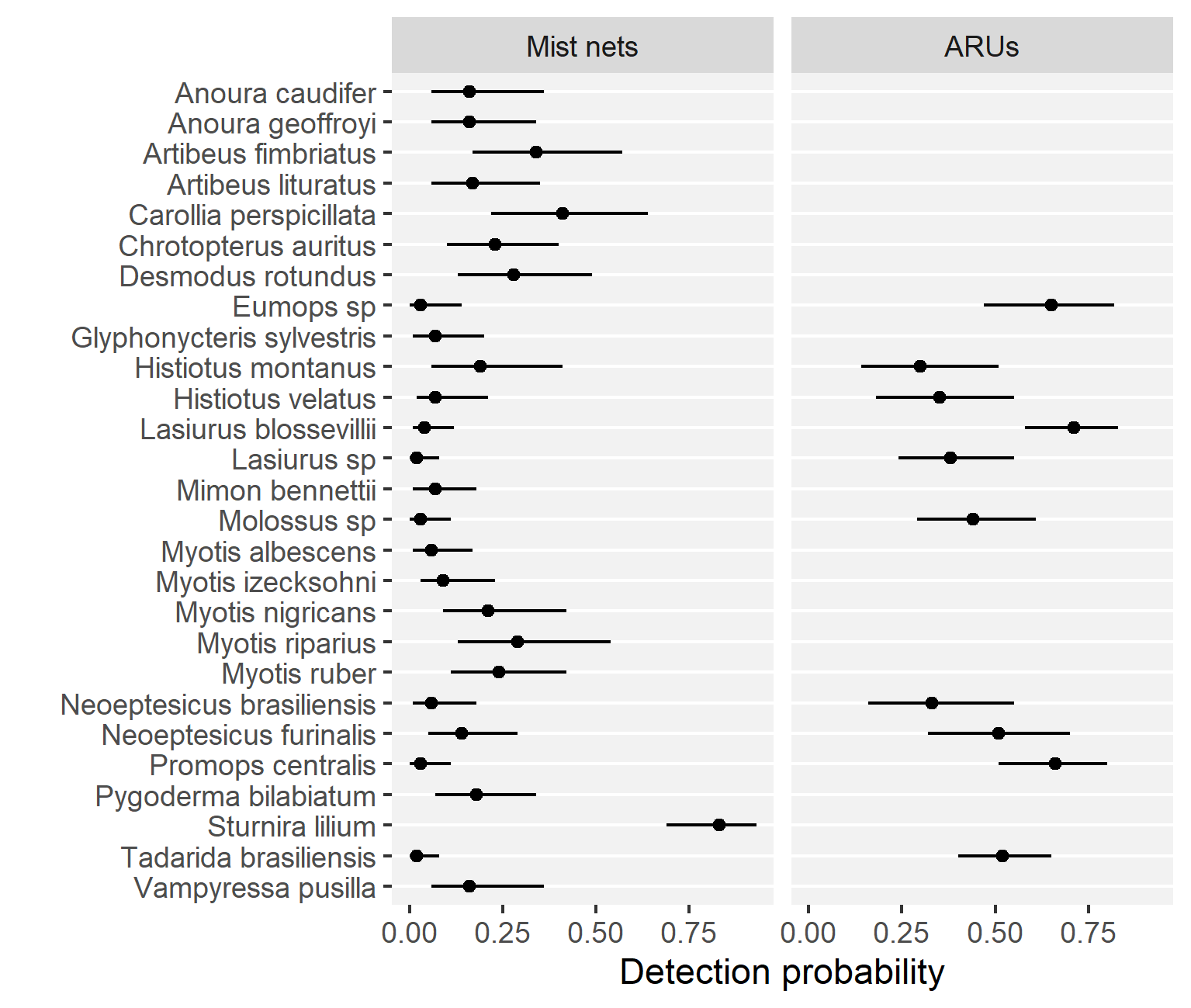


Figure S2. Species-specific detection probabilities for mist net (left) and Acoustic Recording Units (ARUs; right) surveys. Posterior mean estimates (dots) and 95% credible intervals (error bars) are derived from a Bayesian Phylogenetic Occupancy Model (POM).

**REFERENCES**

Alvares, C. A., Stape, J. L., Sentelhas, P. C., Leonardo, J., Gonçalves, M., & Sparovek, G. (2014). Köppen’s climate classification map for Brazil. *Meteorologische Zeitschrif*, *22*(6), 711–728. https://doi.org/10.1127/0941-2948/2013/0507

Arias-Aguilar, A., Hintze, F., Aguiar, L. M. S., Rufray, V., Bernard, E., & Pereira, M. J. R. (2018). Who’s calling? Acoustic identification of Brazilian bats. *Mammal Research*, *63*(3), 231–253. https://doi.org/10.1007/S13364-018-0367-Z/FIGURES/6

Bôlla, D. A. S., Ceron, K., Carvalho, F., De Matia, D. L., Luiz, M. R., Panatta, K. A., Pavei, D. D., Mendonça, R. Á., & Zocche, J. J. (2017). MASTOFAUNA TERRESTRE DO SUL DE SANTA CATARINA: MAMÍFEROS DE MÉDIO E GRANDE PORTE E VOADORES. *Tecnologia e Ambiente*, *23*, 61. https://doi.org/10.18616/ta.v23i0.3906

Díaz, M. M., Solari, S., Gregorin, R., Aguirre, L. F., & Barquez, R. M. (2021). *Clave de identificación de los murciélagos neotropicales*. Programa de Conservación de los Murciélagos de Argentina.

Falcão, F., Dodonov, P., Caselli, C. B., dos Santos, J. S., & Faria, D. (2021). Landscape structure shapes activity levels and composition of aerial insectivorous bats at different spatial scales. *Biodiversity and Conservation*, *30*(8–9), 2545–2564. https://doi.org/10.1007/S10531-021-02210-X/TABLES/1

Garbino, G. S. T., Cláudio, V. C., Gregorin, R., Lima, I. P., Loureiro, L. O., Moras, L. M., Moratelli, R., Nascimento, M. C. do, Nogueira, M. R., Novaes, R. L. M., Pavan, A. C., Tavares, V. da C., & Peracchi, A. L. (2024). Updated checklist of bats (Mammalia: Chiroptera) from Brazil. *Zoologia (Curitiba)*, *41*, e23073. https://doi.org/10.1590/S1984-4689.v41.e23073

Hintze, F., Arias-Aguilar, A., Dias-Silva, L., Delgado-Jaramillo, M., Silva, C. R., Jucá, T., Mischiatti, F. L., Almeida, M., Bezerra, B., Aguiar, L. M. S., Ramos Pereira, M. J., & Bernard, E. (2020). Molossid unlimited: Extraordinary extension of range and unusual vocalization patterns of the bat, Promops centralis. *Journal of Mammalogy*, *101*(2), 417–432. https://doi.org/10.1093/jmammal/gyz167

Jung, K., Molinari, J., & Kalko, E. K. V. (2014). Driving Factors for the Evolution of Species-Specific Echolocation Call Design in New World Free-Tailed Bats (Molossidae). *PLOS ONE*, *9*(1), e85279. https://doi.org/10.1371/journal.pone.0085279

López-Baucells, A. (2018). *Field guide to the bats of the Amazon*. Pelagic Publishing.

Loureiro, L. O., Engstrom, M. D., & Lim, B. K. (2020). Single nucleotide polymorphisms (SNPs) provide unprecedented resolution of species boundaries, phylogenetic relationships, and genetic diversity in the mastiff bats (Molossus). *Molecular Phylogenetics and Evolution*, *143*, 106690. https://doi.org/10.1016/j.ympev.2019.106690

Mackenzie, D. I., Nichols, J. D., Lachman, G. B., Droege, S., Royle, J. A., & Langtimm, C. A. (2002). ESTIMATING SITE OCCUPANCY RATES WHEN DETECTION PROBABILITIES ARE LESS THAN ONE. *Ecology*, *83*(8), 2248–2255. https://doi.org/10.1890/0012-9658

Miller, D. A. W., Pacifici, K., Sanderlin, J. S., & Reich, B. J. (2019). The recent past and promising future for data integration methods to estimate species’ distributions. *Methods in Ecology and Evolution*, *10*(1), 22–37. https://doi.org/10.1111/2041-210X.13110

Novaes, R. L. M., Cláudio, V. C., Díaz, M. M., Wilson, D. E., Weksler, M., & Moratelli, R. (2022). Argentinean Myotis (Chiroptera, Vespertilionidae), including the description of a new species from the Yungas. *Vertebrate Zoology 72: 1187-1216*, *72*, 1187–1216. https://doi.org/10.3897/VZ.72.E90958

Reis, N. R., Peracchi, A. L., Pedro, W. A., & Lima, I. P. (2007). *Morcegos do Brasil* (N. R. Reis, Ed.).
